# Supplementary material for: Pokémon GO, Went, Gone…—Physical Activity Level, Health Behaviours, and Mental Well-Being of Game Users: A Cross-Sectional Study
Source: Healthcare (Basel). 2025 Sep 17;13(18):2334. doi: 10.3390/healthcare13182334 (PMC12469678; doi:10.3390/healthcare13182334)
Supplement: Supplementary file 1 [file healthcare-13-02334-s001.zip › healthcare-3781106-supplementary.pdf]

Supplementary Materials – Custom survey

Section A – Demographics and sports participation

1. Gender
□ Female □ Male

2. Age (years)
Open response

3. Place of residence
□ Village □ Town ≤ 50,000 inhabitants □ Town 51,000–100,000 inhabitants □ Town 101,000–200,000 inhabitants □ City > 200,000 inhabitants

4. Educational level
□ Primary □ Secondary school □ Technical □ High school □ Higher education

5. Occupational status
□ Student □ Unemployed □ Employed □ Retired

6. Do you participate in sports?
□ Yes □ No

7. Type(s) of sport practiced (multiple choice possible)
□ Team sports (e.g., football, volleyball, basketball)
□ Swimming □ Martial arts □ Athletics □ Cycling □ Other: ______

8. How many times per week do you train?
□ 1 □ 2 □ 3 □ 4 □ 5 □ 6 □ 7

9. Average hours of sport per week
Open response

10. For how many years have you been practicing sport?
Open response

11. How do you assess your overall health?
□ Very good □ Good □ Average □ Poor □ Very poor

Section B – Pokémon GO-related questions

1. Since when have you been playing Pokémon GO?
   Open response

2. What motivated you to start playing Pokémon GO? (multiple choice possible)
   □ I am a Pokémon fan
   □ To be more physically active
   □ Because my friends started playing
   □ To meet new people
   □ Because it became trendy
   □ Other: ______

3. How many days per week do you play Pokémon GO?
   □ 1 □ 2 □ 3 □ 4 □ 5 □ 6 □ 7

4. How many hours per day do you usually play Pokémon GO?
   Open response

5. Do you believe you spend a lot of time playing Pokémon GO?
   □ Yes □ No

6. Has your playing time changed over time?
   □ I play more than before □ I play less than before □ I play about the same

7. Would lack of updates/novelties cause you to stop playing?
   □ Yes □ No

8. Do you play any other mobile game that requires outdoor physical activity?
   □ Yes □ No

9. Where do you usually play Pokémon GO? (multiple choice possible)
   □ Around home/neighbourhood
   □ On the way to school/work/university
   □ In parks/green areas
   □ Everywhere possible
   □ Other: ______

10. How do you usually play Pokémon GO? (multiple choice possible)
    □ Walking □ Cycling □ Running
    □ Driving a car □ Using public transport
    □ Other: ______

11. Do you walk more because of playing Pokémon GO?
□ Yes □ No □ Similar as before

12. Do you use public or private transportation less often if you have the time and opportunity to play Pokémon GO “on the way” to your destination?
□ Yes □ No

13. Have you met new friends thanks to playing Pokémon GO?
□ Yes □ No

14. Are your social relationships more diverse because of playing Pokémon GO?
□ Yes □ No

15. Do you prefer to play Pokémon GO alone or with others?
□ Alone □ With family □ With friends □ With people met through the game
□ Other: ______

16. What aspect has Pokémon GO influenced most positively in your case?
□ Physical activity □ Social relationships □ Overall mood □ Other: ______

17. Have you ever given up any physical activity in favor of Pokémon GO? (multiple choice possible)
□ No □ Running □ Team sports □ Swimming □ Gym □ Other: ______

18. Have you experienced pain or discomfort associated with playing Pokémon GO? (multiple choice possible)
□ No
□ Cervical spine □ Lumbar spine □ Shoulder
□ Wrist □ Fingers □ Lower limbs
□ General fatigue/overuse □ Other: ______

19. Have you ever felt fatigued because of playing Pokémon GO?
□ Yes □ No □ Not noticed

20. Have you ever felt relaxed thanks to playing Pokémon GO?
□ Yes □ No □ Not noticed

21. Have you ever knowingly trespassed on private property while playing Pokémon GO?
□ Yes □ No

22. At what time of day do you most often play?

□ Morning □ Afternoon □ Evening/night □ No preference

23. Have you ever sacrificed sleep because of playing Pokémon GO?

□ Yes □ No

24. Have you ever found yourself in a health-threatening situation due to inattention caused by playing Pokémon GO?

□ Yes □ No

25. Has Pokémon GO influenced your motivation for study/work?

□ Yes, I am more motivated □ Yes, I am less motivated □ No effect

26. How much money do you spend on Pokémon GO monthly?

Open response

27. Which operating system do you use to play Pokémon GO?

□ iOS □ Android □ Both

28. Do you believe you are addicted to playing Pokémon GO?

□ Yes □ No
